# Characterization of Iron Oxide Nanotubes Obtained by Anodic Oxidation for Biomedical Applications—In Vitro Studies

**DOI:** 10.3390/ma17153627

**Published:** 2024-07-23

**Authors:** Rita de Cássia Reis Rangel, André Luiz Reis Rangel, Kerolene Barboza da Silva, Ana Lúcia do Amaral Escada, Javier Andres Munoz Chaves, Fátima Raquel Maia, Sandra Pina, Rui L. Reis, Joaquim M. Oliveira, Ana Paula Rosifini Alves

**Affiliations:** 1 São Paulo State University (UNESP), School of Engineering, Ilha Solteira 15385-007, Brazil; rita.r.rangel@unesp.br (R.d.C.R.R.); al.rangel@unesp.br (A.L.R.R.); 2 São Paulo State University (UNESP), School of Engineering and Sciences, Guaratinguetá, São Paulo 01049-010, Brazil; kerolene.barboza@unesp.br (K.B.d.S.); ala.escada@unesp.br (A.L.d.A.E.); 3 Intelligent System Research Group, Faculty of Engineering, Corporación Universitaria Comfacauca-Unicomfacauca, Popayán 190003, Colombia; jmunoz@unicomfacauca.edu.co; 4 3B’s Research Group, I3Bs—Research Institute on Biomaterials, Biodegradables and Biomimetics, University of Minho, AvePark, Parque de Ciência e Tecnologia, Zona Industrial da Gandra, Barco, 4805-017 Guimarães, Portugal; raquel.maia@i3bs.uminho.pt (F.R.M.); sandra.pina@i3bs.uminho.pt (S.P.); rgreis@i3bs.uminho.pt (R.L.R.); miguel.oliveira@i3bs.uminho.pt (J.M.O.); 5 ICVS/3B’s—PT Government Associated Laboratory, 4710-057 Guimarães, Portugal

**Keywords:** biodegradable biomaterials, surface modification, biodegradable metal

## Abstract

To improve the biocompatibility and bioactivity of biodegradable iron-based materials, nanostructured surfaces formed by metal oxides offer a promising strategy for surface functionalization. To explore this potential, iron oxide nanotubes were synthesized on pure iron (Fe) using an anodic oxidation process (50 V–30 min, using an ethylene glycol solution containing 0.3% NH_4_F and 3% H_2_O, at a speed of 100 rpm). A nanotube layer composed mainly of α-Fe_2_O_3_ with diameters between 60 and 70 nm was obtained. The effect of the Fe-oxide nanotube layer on cell viability and morphology was evaluated by in vitro studies using a human osteosarcoma cell line (SaOs-2 cells). The results showed that the presence of this layer did not harm the viability or morphology of the cells. Furthermore, cells cultured on anodized surfaces showed higher metabolic activity than those on non-anodized surfaces. This research suggests that growing a layer of Fe oxide nanotubes on pure Fe is a promising method for functionalizing and improving the cytocompatibility of iron substrates. This opens up new opportunities for biomedical applications, including the development of cardiovascular stents or osteosynthesis implants.

## 1. Introduction

The conventional approach involving the utilization of metallic materials in biomedical applications prioritizes not only mechanical properties but also demands high resistance to corrosion within the body. However, a novel category of biodegradable materials, particularly biodegradable metals, has emerged as a viable alternative for medical implant devices, challenging the traditional paradigm [1,2]. Biodegradable or absorbable materials undergo gradual corrosion and degradation in the body’s environment. These materials are engineered to decompose into non-toxic byproducts that can be metabolized or assimilated by cells or tissue. Once they have fulfilled their function within the body, they completely dissolve and are eliminated through the patient’s normal metabolic processes [2,3,4,5].

The primary biodegradable metals proposed to address the limitations of permanent implant materials include iron (Fe), magnesium (Mg), and zinc (Zn) [6,7,8]. Fe and its alloys have been extensively studied as potential candidates for biodegradable stents due to their favorable biocompatibility and mechanical properties [9,10]. These Fe-based implants can demonstrate high strength, with values reaching up to 1450 Mpa, along with high ductility, with elongation values of up to 80%. The exceptional strength of these materials allows for the production of thin struts, while their high ductility facilitates significant plastic deformation, aiding in vascular deployment [11,12,13]. In initial in vivo studies, pure Fe stents were evaluated, revealing good biocompatibility and low neointimal proliferation, comparable to results obtained with standard materials like 316L stainless steel and cobalt-chromium alloys. Additionally, no significant indications of inflammation or local/systemic toxicity were associated with Fe. However, despite its considerable promise, the results also indicated that the in vivo degradation rate of pure Fe was very low, potentially failing to meet clinical requirements [14,15,16,17]. Another limitation of Fe implants is their ferromagnetic characteristic, which impairs compatibility with some imaging devices, such as magnetic resonance imaging [18].

Various strategies, including alloying [18,19,20,21,22,23], processing [24,25,26,27,28,29,30], and surface modifications [31,32,33,34,35,36], can be utilized to enhance the mechanical properties and corrosion rate of pure iron (Fe). One approach to achieving a balance between biomechanical performance and biological function involves creating porous structures on the surface of the implant material. By generating nanostructures on the material’s surface, properties such as osteoinduction, bioabsorption, biocompatibility, and biodegradability can be attained, enhancing the interaction of the implant with cells [37,38,39].

The electrochemical technique known as anodizing, or anodic oxidation, is a well-established method for creating nanostructured surfaces on metals. This process involves the adsorption of oxygen from the electrolyte solution onto the metal substrate, leading to the formation of an oxide layer. Depending on various parameters such as material composition, potential, and electrolyte, the outcome can range from forming nanopores to creating ordered nanotubes [40,41]. Because of their porous structure, nanotubes can function as drug reservoirs capable of being loaded with various medications depending on their intended application. Additionally, they can also affect the degradation behavior of the material [42].

The use of Iron oxide nanotubular arrays In biomaterial applications has hardly been investigated in the literature. Therefore, to consider their biomedical application, it is essential to study the influence of nanostructures on the degradation process and the biocompatibility of the Fe substrate.

The novelty of this work is the development of an experimental methodology that allows the preparation of iron nanotubes on the pure iron surface by anodic oxidation for biomedical applications. Our goal was to obtain a hydrophilic surface that presents a better biological response associated with better surface properties. The morphology and crystalline structure of the nanotubes were characterized, and in vitro studies using a human osteosarcoma cell line (SaOs-2 cells) were performed by culturing the cells on the surface-modified materials to assess the effect on cellular responses and substrate cytocompatibility.

## 2. Materials and Methods

### 2.1. Experimental Procedure

Ingots of iron (>99.98% purity, Sigma-Aldrich, St. Louis, MO, USA) were fabricated by melting the metals in a water-cooled copper crucible using an arc furnace under an argon atmosphere (>99.998% purity, White Martins, Guaratinguetá, SP, Brazil). After the melting, the following processing route was used: homogenization heat treatment at 1000 °C for 24 h under vacuum; hot rotary swaging to produce bars with a diameter of 10 mm; heat treatment at 1000 °C for 2 h followed by quenching in water. The quantitative chemical analysis of the bars was determined by an X-ray fluorescence analyzer (XRF, Panalytical, Axios MAX, Almelo, The Netherlands).

The bars were sectioned into discs with a thickness of 3 mm and utilized as substrates for characterization and subsequent surface modification. For the surface treatment, the samples were ground using silicon carbide sandpaper (#220–#1200), cleaned in an ultrasonic bath for 10 min (in water and ethyl alcohol), and dried by hot air.

The anodic oxidation process was used to produce Fe oxide nanotubes at room temperature. The parameters were chosen based on our experience with TiO_2_ nanotubes and previous research investigating the effects of anodic oxidation conditions to achieve an attractive and effective nanostructure [43,44]. In addition, the temperature range for annealing was chosen to maintain the structural integrity of the formed nanotubes [45,46].

**Figure 1:**
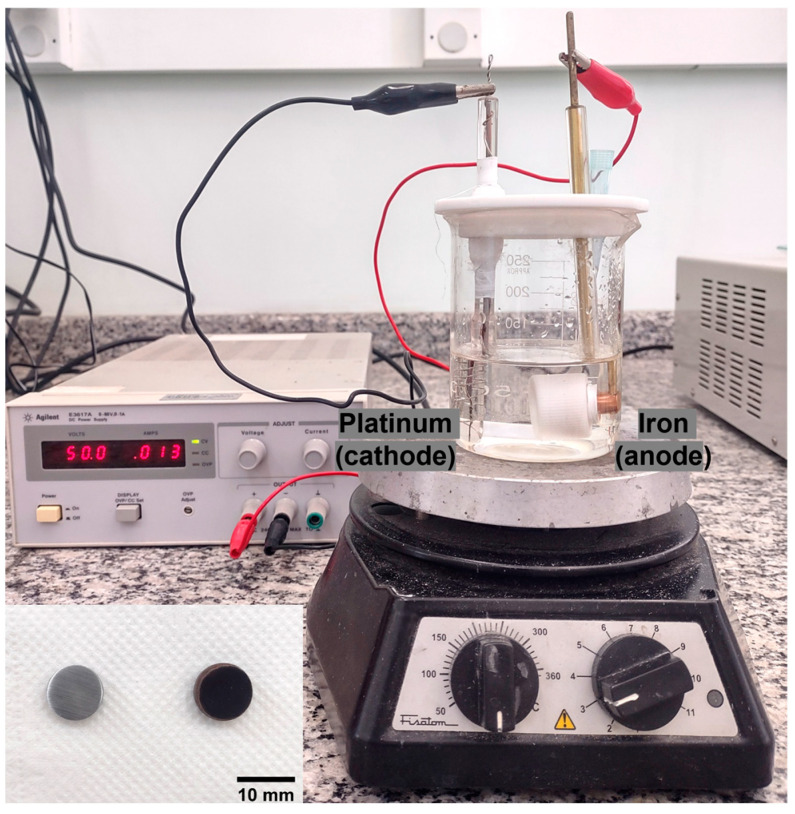
The electrochemical cell is composed of two electrodes, a working electrode (anode) and a platinum counter-electrode (cathode). The images in the bottom left corner show the sample before (left) and after (right) the process of anodic oxidation.

The anodic oxidation was performed in an electrochemical cell consisting of two electrodes—a working electrode (anode) and a platinum counter electrode (cathode)—connected to a voltage source (Figure 1). The electrolyte solution used consisted of ethylene glycol (99.0% purity, Synth, Diadema, Brazil) with 0.3 wt.% of NH_4_F (98.0% purity, Dinâmica, Diadema, Brazil) and 3 vol% DI water. The samples were anodized by applying a constant voltage of 50 V (DC power supply—Agilent E3617A, Santa Clara, CA, USA) for 30 min, with a magnetic stirrer (Fisatom, model 752A, São Paulo, Brazil) at 100 rpm. Subsequently, the samples were annealed at 350 °C and 400 °C for 2 h to observe the transformation of the crystalline structure of Fe oxide. The samples prepared at different temperatures were categorized as Fe NT 350 °C and Fe NT 400 °C, respectively.

### 2.2. Surface Characterization

Optical microscopy (OM, Nikon-Epiphot, Nikon Inc., Melville, NY, USA) was used to evaluate the iron surface before surface treatment. Metallographic samples were carefully ground with 100–2000 grit SiC abrasive papers, polished with colloidal silica, cleaned ultrasonically, and dried in air. They were etched using a 2% Nital solution (2% by volume of 65% PA nitric acid, Qhemis, in 99.5% PA ACS absolute ethanol, Nox Solutions, São Paulo, Brazil) to delineate the grain boundaries. The average grain size was measured from the obtained micrographs with the ImageJ software version 1.54.

The surface morphologies of the samples were characterized by scanning electron microscopy (SEM) (TESCAN/MIRA 3, Brno, Czech Republic). The average dimensions of the nanostructures were also measured using ImageJ image analysis software. The qualitative crystalline structure of the samples was analyzed through X-ray diffraction (XRD) (Panalytical X’Pert, Almelo, The Netherlands) with Cu Kα radiation and produced at 45 kV and 40 mA. The scan was performed at a 2θ range of 5–90° with a 0.02° step size and 5 s per step. The vibrational characteristics were investigated employing Raman spectroscopy (LabRam HR, Horiba Jobin Yvon, Palaiseau, France). The spectra were collected using the line 514 nm of an argon laser as an excitation source.

The samples’ surface was also analyzed utilizing X-ray photoelectron spectroscopy (XPS, Kratos Axis-Supra instrument, Kratos Analytical, Manchester, UK) equipped with an aluminum Kα (Al-Kα) monochromatized radiation X-ray source at 1486.6 eV, with the assistance of ESCApe software (Version 1.4, Kratos Analytical, Manchester, UK). Photoelectrons were collected from a take-off angle of 90° relative to the sample surface. The chemical composition of samples’ surfaces was examined in a Constant Analyser Energy mode (CAE) (Kratos Analytical) with 160 eV pass energy for survey spectra and 20 eV pass energy for high-resolution spectra and an emission current of 10 mA for all the acquisitions. The high-resolution spectra were performed for different elements to determine the relative composition of the sample’s surface. Charge referencing was performed by setting the lower binding energy C 1s photopeak at 285.0 eV, which is related to the C 1s hydrocarbon peak.

Contact angle measurements were carried out using goniometer equipment (DSA 100 model, Krüss, Hamburg, Germany) to evaluate the wettability of the Fe surfaces. For this, 10 μL of deionized water was used as the test liquid.

### 2.3. In Vitro Cell Studies

A human osteosarcoma cell line, SaOs-2 cells, was obtained from CLS Cell Lines Service GmbH (Eppelheim, Germany) and used for screening the possible cytotoxicity of Fe, Fe NT 350 °C, and Fe NT 400 °C discs. For that, 200,000 cells were seeded on the top of each disc and kept in culture in Dulbecco’s modified eagle medium (DMEM, Sigma-Aldrich) supplemented with 10% *v*/*v* fetal bovine serum (FBS; Alfagene, Carcavelos, Portugal) and 1% *v*/*v* antibiotic/antimycotic (Life Technologies, Carlsbad, CA, USA) for 14 days at 37 °C under a humidified atmosphere of 5% *v*/*v* CO_2_. The culture media was renewed twice a week. On days 1, 3, 7, and 14 days of the culture, cells’ metabolic activity and morphology were analyzed.

The high-resolution field emission scanning electron microscope (HR-SEM, AURIGA COMPACT, Oberkochen, Germany) with an accelerating voltage of 5 kV was utilized to examine the discs and observe cell morphology during cultivation. Before analysis, the samples were fixed in formalin 10%, rehydrated with different ethanol concentrations (50, 70, 90, and 100% *v*/*v*) for 15 min, and placed in hexamethyldisilazane (Sigma-Aldrich) overnight at room temperature (RT) to dry. Before SEM analysis, the discs were sputter coated with platinum (Pt) using a Leica EM ACE600 coater (Leica Microsystems, Mannheim, Germany). The disc surfaces were then semi-quantitatively analyzed using energy-dispersive spectrometry (EDS, Oxford Instruments, Abingdon, UK) at 20.0 eV. The SEM image colorization was performed using MountainsLab^®^ software version 10.2 (Digital Surf Co., Besançon, France).

SaOs-2 cells’ metabolic activity was assessed using the AlamarBlue^®^ reagent (Biorad, Hercules, CA, USA). At each time point, cells were incubated with 20% *v*/*v* of reagent in the culture medium for 3 h at 37 °C. The supernatant was then transferred to a new 96-well tissue culture plate, and fluorescence measurements were carried out using a microplate reader (Biotek Synergy HT, Winooski, VT, USA) at 530/20 nm (excitation) and 590/35 nm (emission). AlamarBlue^®^ reagent in medium served as a blank.

SaOs-2 cells’ adhesion and spreading were studied through F-actin staining. For that, cells were washed with phosphate-buffered saline (PBS, Sigma-Aldrich), fixed with 10% Neutral Buffered Formalin (Thermo Fisher Scientific, Waltham, MA, USA) for 20 min, and permeabilized for 5 min with 0.1% *v*/*v* Triton X-100 (Sigma-Aldrich) in PBS. Afterward, cells were incubated for 30 min with 1% bovine serum albumin (BSA, Sigma-Aldrich) in PBS to reduce nonspecific background staining. Finally, F-actin filaments were stained with Alexa Fluor 488 Phalloidin (Thermo Fisher Scientific, 1:40), and nuclei were counterstained with 4,6-Diamidino-2-phenyindole, dilactate solution (DAPI, 300 nM, ThermoFisher Scientific). Samples were analyzed by confocal microscopy (Leica TCS SP8, Mannheim, Germany).

Statistical analyses were carried out using GraphPad Prism 8. First, the Shapiro–Wilk test was performed to assess data normality. The nonparametric tests, the Kruskal–Wallis test followed by the Dunn’s test, were used to analyze the metabolic activity results. The critical value of statistical significance was *p* < 0.05. Data are cited as mean ± standard error of the mean.

## 3. Results

The chemical composition of the bars of iron after processing was verified. A relatively low concentration of other elements, such as Si (0.18% by weight), Al (1.04% by weight), Na (0.10% by weight), and S (0.08% by weight), was identified. The detection of aluminum was attributed to the use of an alumina crucible during the heat treatment of the iron bars, carried out after the melting and forging processes.

**Figure 2:**
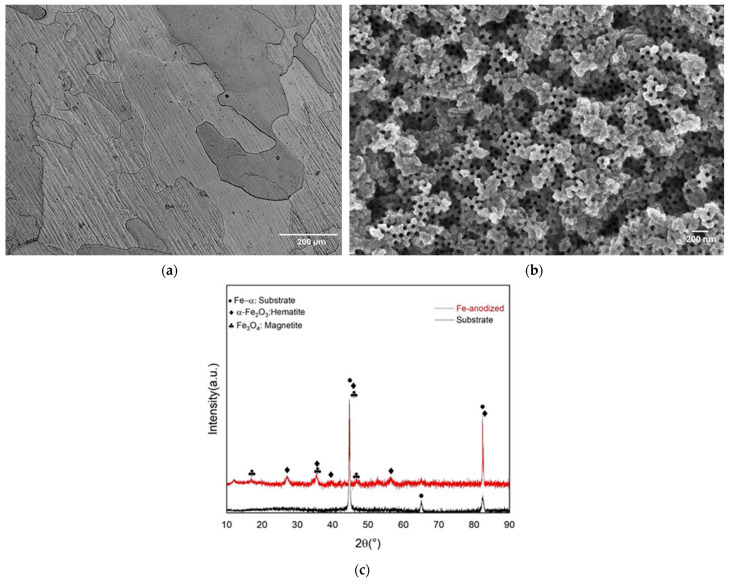
(**a**) Optical micrograph of the pure Fe sample after processing, (**b**) Scanning electron microscopy (SEM) images of the nanotubes layer after anodic oxidation before annealing, and (**c**) X-ray diffraction (XRD) spectrum of both conditions.

The single-phase microstructure (the phase was determined by XRD) was verified in the substrate surface using optical microscopy as shown in Figure 2a. An average grain diameter of 121 μm was observed [47,48]. After anodic oxidation, it was possible to observe the formation of a nanotube layer on the top-view surface image (Figure 2b).

X-ray diffraction (XRD) analysis was utilized to identify the phases present on the surfaces before and after surface treatment (Figure 2c). Typical peaks of ferrite α phase (bcc) were observed on the substrate surface, while new peaks corresponding to crystalline hematite (α-Fe_2_O_3_) and magnetite (Fe_3_O_4_) phases were detected after anodic oxidation and annealing. The ferrite α phase is commonly found in iron and its alloys [19,48]. The development of these new phases associated with the morphologies of the surfaces formed leads to the conclusion that anodic oxidation successfully produced the iron oxide layer.

**Figure 3:**
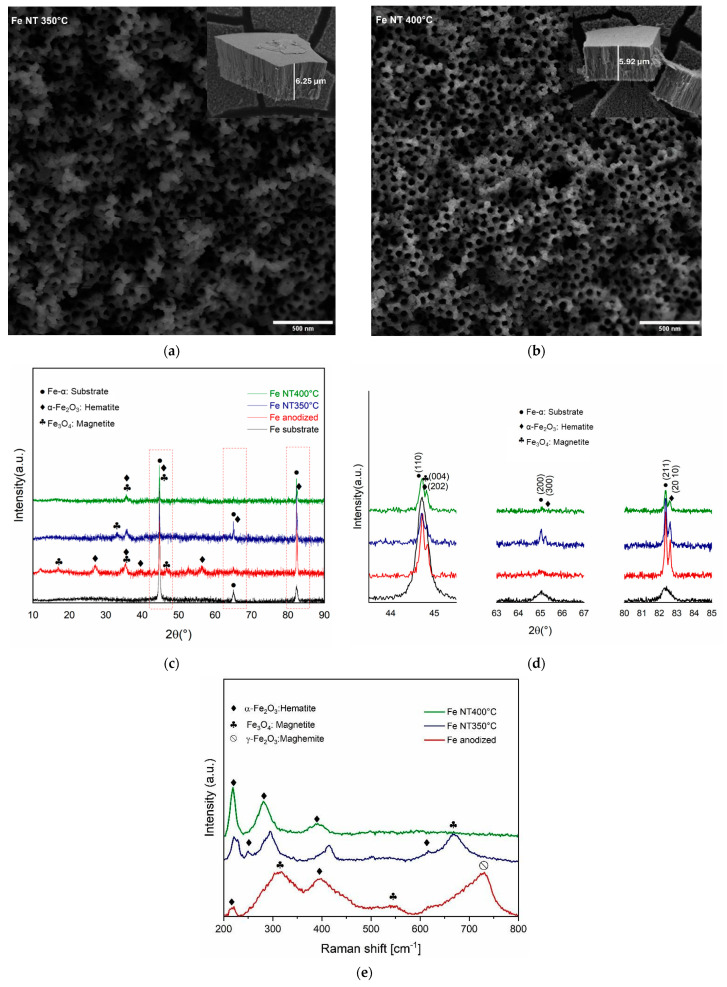
SEM micrographs showing the surface morphology of Fe samples anodized and annealed at (**a**) 350 °C and (**b**) 400 °C. The scale bar for the layer thickness in both (**a**) and (**b**) is 20 µm. (**c**) XRD patterns of the Fe sample after anodic oxidation and annealing at 350 °C and 400 °C. (**d**) Magnified view of the regions of interest marked with dashed red lines in (**c**). (**e**) Raman spectra for the studied conditions.

In Figure 3, it is possible to observe the iron surface after anodic oxidation following annealing at (a) 350 °C (Fe NT 350 °C) and (b) 450 °C (Fe NT 400 °C). In the figure inserts, small areas of the surfaces were vigorously scratched with tweezers to detach the nanostructured layer and allow measurement of the layer thickness. Fe NT 350 °C samples exhibited a uniform tubular nanostructure, with most of the pores being open at the top of the layer. Based on the micrographs, the average pore diameter and layer thickness of the samples were estimated to be 70.02 ± 1.13 nm and 6.25 ± 0.18 µm, respectively. For the Fe NT 400 °C samples, the average pore diameter and layer thickness were estimated to be 60.46 ± 4.74 nm and 5.92 ± 0.13 µm, respectively. These observations were derived from measurements carried out using ImageJ software. For each temperature condition, the inner diameters of the nanotubes and the layer thicknesses were measured. From these data, the mean values and their respective standard deviations were calculated. The results are comparable to those found in the research on the anodic growth of highly ordered nanotubes of iron oxide in ethylene glycol/NH_4_F electrolytes, where a layer thickness of up to 7.5 µm was observed, with variations in pore diameters (up to 100 nm), depending on the applied potential [49]. The structure of nanotube arrays, including their diameter, thickness, and surface morphology, is critical for the drug reservoir system.

Studies have shown that increasing the layer thickness generally leads to a higher total surface area. This results in the formation of nanotubular matrices that can have a superior drug-loading capacity [42,50]. Furthermore, the structure of nanotubes may exhibit advantageous cellular functions in terms of cell adhesion, proliferation, and migration. Several studies have shown that nanotubes with diameters ranging from 30 nm to 70 nm can exhibit better cellular functioning compared to non-anodized metallic substrates [51,52,53].

Structural characterization obtained by X-ray diffraction (XRD) (Figure 3c) exhibited the typical crystalline peaks of the Fe-α phase for the substrate and hematite (α-Fe_2_O_3_) and magnetite (Fe_3_O_4_) phases for anodized and annealed conditions at 350 °C and 400 °C. Among them, hematite presented the main phase characterized by the diffraction planes (012), (113), and (116) located at ~27°, 39.7°, and ~55.4°, respectively. It was also observed that the Fe NT350 °C presented higher crystallinity in comparison to the others (Figure 3d).

Vibrational characteristics were obtained using Raman spectroscopy (Figure 3e). The results are in agreement with those observed for the XRD, as hematite was observed as the representative phase of the nanostructure. This phase belongs to the $D_{3d}^{6}\;$space group, and seven well-established vibration modes are expected in the Raman spectrum, two $A_{g}^{1}$ modes (225 and 498 cm^−1^) and five E_g_ modes (247, 293, 299, 412, and 613 cm^−1^) [54,55]. Among these, the spectra of the anodized and heat-treated samples showed the presence of peaks at around 219 cm^−1^ (A_1g_), 247 cm^−1^ (E_g_), 290 cm^−1^ (E_g_), 400 cm^−1^ (E_g_) and 615 cm^−1^ (E_g_). Magnetite, considered to be in a smaller proportion in the nanostructure formed, also in agreement with the XRD (Figure 3d), belongs to the space group $O_{h}^{7}$, and five Raman bands are expected: three T_2g_, one E_g,_ and one A_1g_ [54,55].

There are some divergences in the values reported for peak indexing [56]. However, based on the literature, it has been established that peaks at ~310 cm^−1^ (T_2g_), 554 cm^−1^ (T_2g_), and 672 cm^−1^ (A_1g_) are presented [57,58]. From the spectra, the peaks were identified mainly as magnetite (Fe_3_O_4_) and hematite (α-Fe_2_O_3_), alongside the possible presence of maghemite (γ-Fe_2_O_3_), which is considered a transitional phase between magnetite and hematite [56,59].

X-ray photoelectron spectroscopy (XPS) analysis was used to evaluate the chemical composition of the layer formed. The XPS wide survey results are summarized in Table 1. A very low concentration of Fe on untreated surfaces can be observed, mostly due to organic contamination, as the carbon amount was almost 80% of the atomic concentration. After anodic oxidation, this scenario changed, revealing a Fe atomic concentration that was at least ten times higher for both treated conditions. Two other possible contaminants were verified after treatment: copper, most probably for the electrode supports, and fluorine (F) from the electrolyte, both during the anodization process of the samples. The amount of F was in agreement with what has been previously reported for annealed Fe nanotubes produced with ammonium fluoride [49]. It is noticeable that an increase in annealing temperature results in a decrease in the F atomic concentration.

A high-resolution XPS analysis was performed on the samples to obtain detailed information about the chemical composition and electronic structure of the surfaces (Table 2).

For untreated surfaces, the results confirm that most of the Fe was in the form of Fe III (711.2; 718.6; 718.8; 724.6 eV) [60,61,62]. After treatment, the amount of the Fe II (719.9) [63,64] components increased, yet the predominance of the Fe III form is still verified. A new binding energy peak was observed at 719.5 eV, which is commonly linked to Fe in a low-spin state and a distorted octahedral coordination environment, particularly in Fe oxide or hydroxide compounds (Fe III), such as goethite (FeO(OH)), akaganeite (β-FeOOH), or certain forms of ferric oxyhydroxide [65,66]. It should be cited that the percentage of these components was less significant in the samples annealed at 400 °C. This result suggests that a higher annealing temperature has a stabilizing effect on the oxide layer formed.

Another interesting evidence of the higher presence of oxide after treatment is the change in the Oxygen (O) bonds proportion. It is generally expected that O atoms in inorganic molecules would have a lower binding energy compared to those in organic molecules. The O 1s peak at 529.9 eV, prevalent after treatment, is often linked to metal-oxygen bonds, such as those found in metal oxides [67,68], while the O 1s peaks at 531.2 eV and 532.2 eV are associated with C-O-C or C=O bonds [69,70].

The analysis also indicates that F is predominantly presented in an inorganic form. The F 1s XPS peaks at 683.7 eV and 685.3 eV are typically associated with the presence of F in inorganic compounds, such as metal fluorides or fluorinated oxides [71,72,73]. Since the Fe peaks do not indicate Fe/F bonding, these compounds are most likely formed with copper, which is reinforced by the presence of the Cu 2p3/2 peaks at 936.9 and 952.9 eV, attributed to CuF_2_ [74].

Wettability is an important surface property associated with surface morphology. Studies with TiO_2_ nanotubes have found that hydrophilic surfaces tend to enhance cell adhesion, proliferation, differentiation, and bone mineralization compared to hydrophobic surfaces [75].

**Figure 4:**
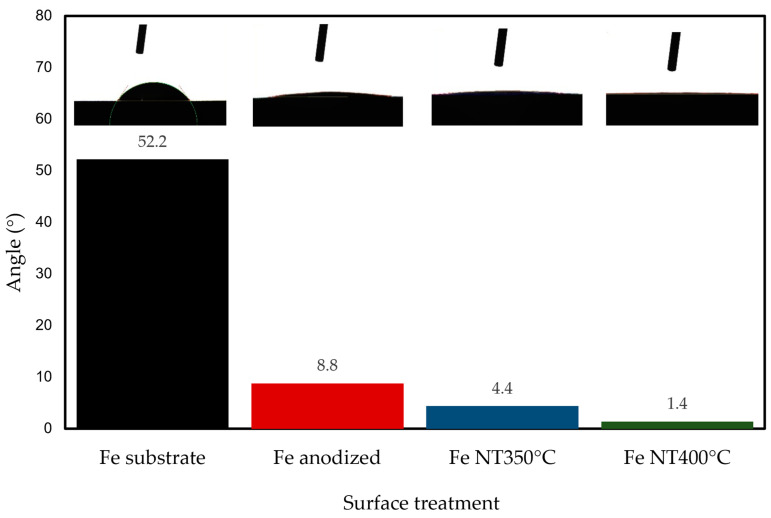
Contact angle of the iron surface (substrate), after anodic oxidation and annealing at 350 °C and 400 °C.

Values of contact angle >90° indicate a hydrophobic surface and < 90° indicate a hydrophilic surface, while an angle less than 5° indicates a superhydrophilic surface [76]. In this study, the contact angle measurements demonstrated that the wettability was considerably enhanced following the surface modification process (Figure 4). The contact angle decreased from 52.2° to 8.8° after anodic oxidation and decreased even further after annealing at 350 °C and 400 °C, resulting in contact angles of 4.4° and 1.4°, respectively. These well-organized tubular nanostructure surfaces are considered superhydrophilic. This can have an important consequence on cell adhesion to the substrate containing the nanotube, as it has been reported that cells tend to attach better to hydrophilic surfaces as compared to hydrophobic ones [77,78,79].

**Figure 5:**
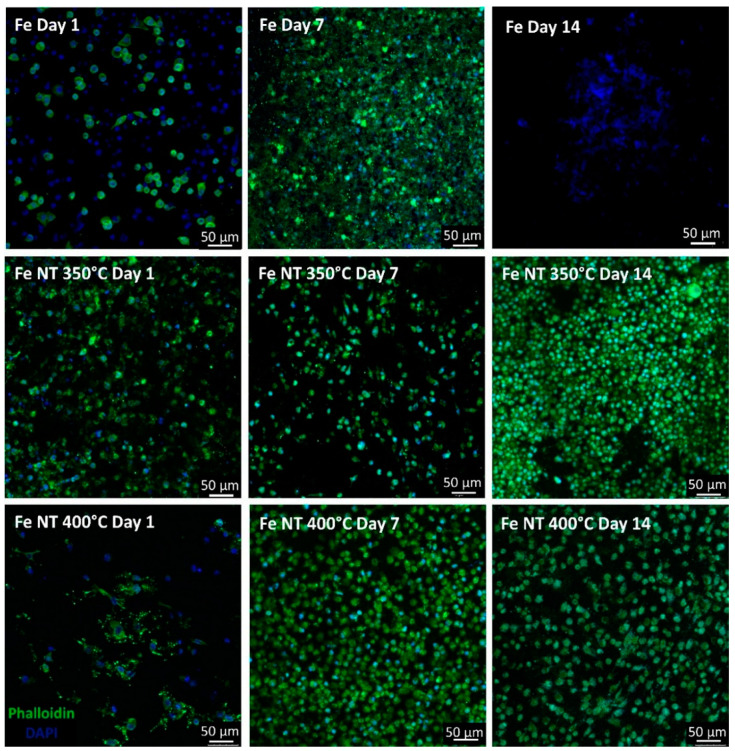
F-actin staining of SaOs-2 cells along 14 days of culture grown in different Fe samples. F-actin staining of cells grown in discs to assess cellular adhesion and spread along 14 days of culture. Cells’ F-actin was stained with phalloidin (in green), and the nuclei were counterstained with DAPI (in blue) (scale bar = 50 µm).

Cells’ F-actin was stained with phalloidin to assess cellular adhesion and spread to discs developed during culture (Figure 5). On Day 1, cells adhered to all conditions; however, the cytoskeleton was more developed over surfaces containing nanotubes (Fe NT 350 °C). SaOs-2 cells cultivated onto the Fe NT 400 °C presented some damaged cytoskeleton.

On day 7, SaOs-2 cells cultured onto the surface of Fe discs were able to attach and proliferate during 7 days of culture. For Fe NT 350 °C samples, cell proliferation decreased compared to Fe and Fe NT 400 °C, which was reverted over time, showing that cells could recover and continue proliferating until Day 14.

For cells grown on Fe disks, on day 14, only cell nuclei were noticeable, and no F-actin filaments were visibly stained. For Fe NT 350 °C, cell proliferation increased, while at Fe NT 400 °C, the cells were larger and spread out.

Therefore, when we compare the surfaces containing NTs, it was observed that on day 7, the number of cells present on the surface of Fe NT 400 °C was already greater than on the surface of Fe NT 350 °C, showing that this was the best condition, since for the Fe NT 350 °C sample, this similarity in cell quantity was only achieved on day 14.

**Figure 6:**
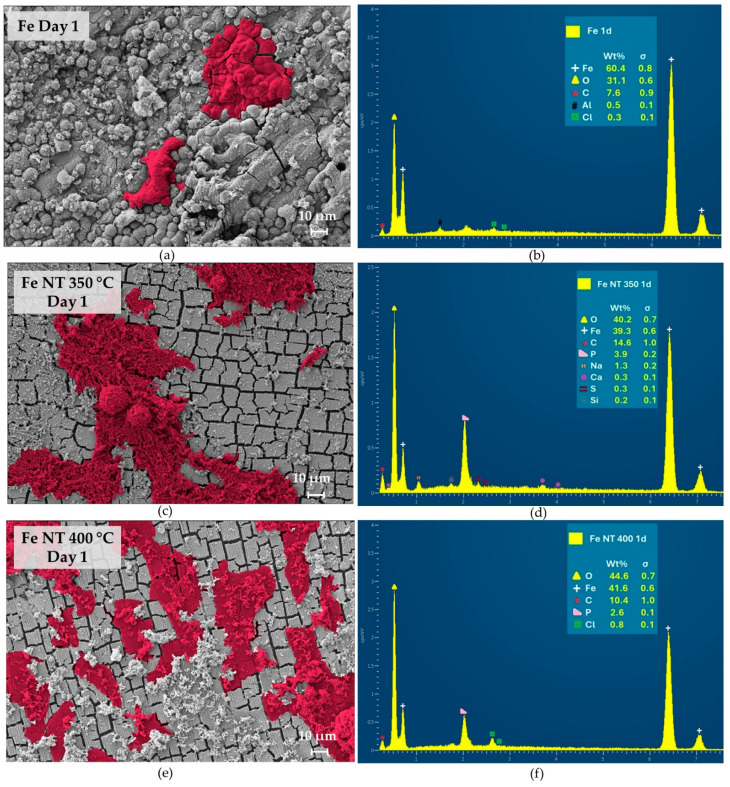
HR-SEM images and EDS analysis of the discs surfaces on day 1 of in vitro culture: (**a**) HR-SEM image and (**b**) EDS analysis of Fe control, (**c**) HR-SEM image and (**d**) EDS analysis of Fe NT350 °C, (**e**) HR-SEM image and (**f**) EDS analysis of Fe NT400 °C.

**Figure 7:**
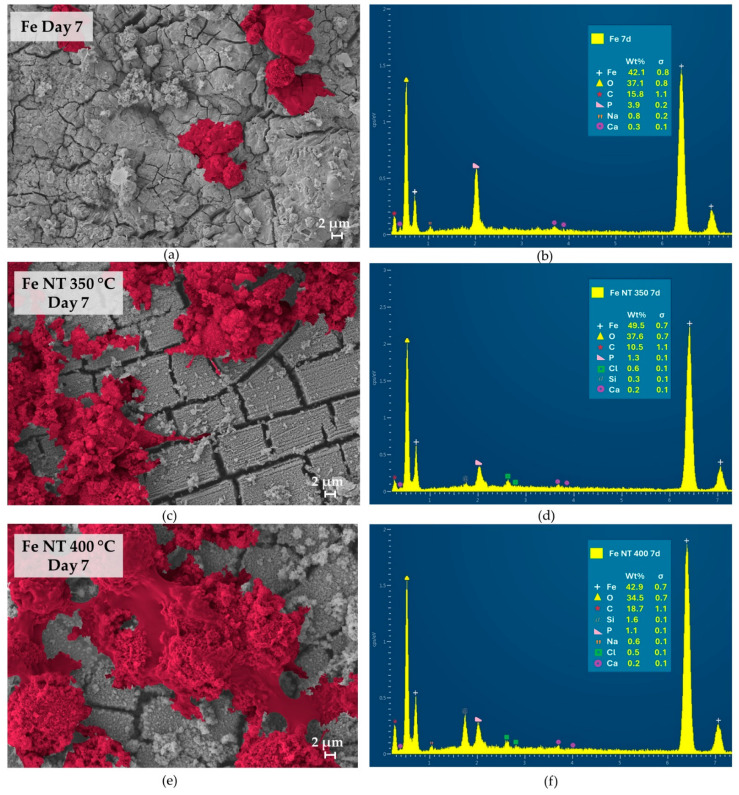
HR-SEM images and EDS analysis of the discs surfaces on day 7 of in vitro culture: (**a**) HR-SEM image and (**b**) EDS analysis of Fe control, (**c**) HR-SEM image and (**d**) EDS analysis of Fe NT350 °C, (**e**) HR-SEM image and (**f**) EDS analysis of Fe NT400 °C.

**Figure 8:**
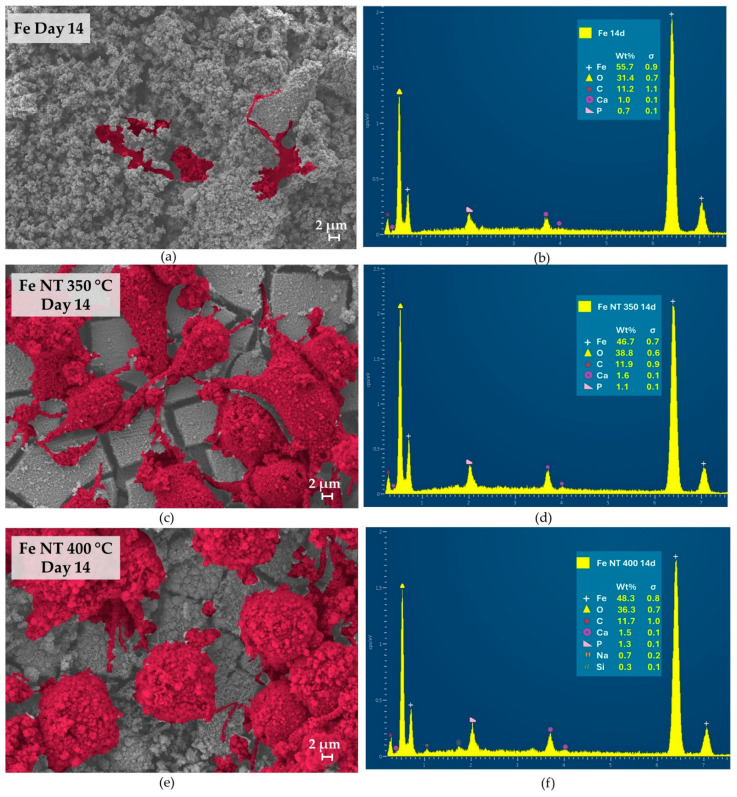
HR-SEM images and EDS analysis of the discs surfaces on day 14 of in vitro culture: (**a**) HR-SEM image and (**b**) EDS analysis of Fe control, (**c**) HR-SEM image and (**d**) EDS analysis of Fe NT350 °C, (**e**) HR-SEM image and (**f**) EDS analysis of Fe NT400 °C.

The topography of developed discs and the morphology of cultured SaOs-2 cells were examined using high-resolution scanning electron microscopy (HR-SEM). Meanwhile, energy-dispersive X-ray spectroscopy (EDS) was used to semi-quantitatively analyze the surface of the observed samples (Figure 6, Figure 7 and Figure 8). Aiming to highlight the differences in cell development on the studied surfaces, the image analysis software Mountains was used to colorize the cells.

The cell morphology and proliferation differed between the treated and untreated surfaces. EDS analysis verified the presence of Ca, P, and O on the sample surfaces at different stages of cell culture, depending on the presence of nanotubes. These elements are important indicators of surface biomineralization, which was influenced by the treatment.

Starting from Day 1, it was possible to observe a superior attachment of SaOs2-cells over treated surfaces, as observed in Figure 6c,e. The EDS of the surface of the pure iron sample (Fe control) did not show the presence of the elements Ca and P (Figure 6b). On the other hand, their presence was observed on Fe NT 350 °C surface (Figure 6d) and Fe NT 400 °C surfaces (Figure 6f), which indicates that biomineralization was accelerated by the nanotube formation. Such behavior was previously verified by our group on surfaces with titanium nanotubes [53,80], thus indicating that the morphology of the nanostructured surfaces would be the main reason for the superior attachment and proliferation of SaOs-2 cells and better cell spread.

The differential behavior between anodized and non-anodized surfaces in terms of cell morphology has been previously observed in titanium treated surfaces [81]. The present results indicate another interesting outcome dependent on the annealing temperature. Starting from day 7, samples annealed at 400 °C (Figure 7e) exhibited a more spread cell morphology, with more prominent cytoplasm development and broad pseudopods. In contrast, samples annealed at 350 °C (Figure 7c) displayed cells that were less developed around the nucleus, presenting a thinner cell shape. Also on Day 7, the presence of the three elements (Ca and P) was verified for all surfaces (Figure 7b,d,f). Muhammad Rabeeh (2022) verified the same type of behavior for porous pure iron surfaces by gold sputtering for degradable implants, with its bioactivity and osseointegration characteristics of the surface improving [82].

For Day 14, the SEM results corroborated the results obtained in the fluorescence images, indicating that for Fe NT 350 °C, cell proliferation increased; nevertheless, in Fe NT 400 °C, the cells were larger and spread out (Figure 8a,c,e).

**Figure 9:**
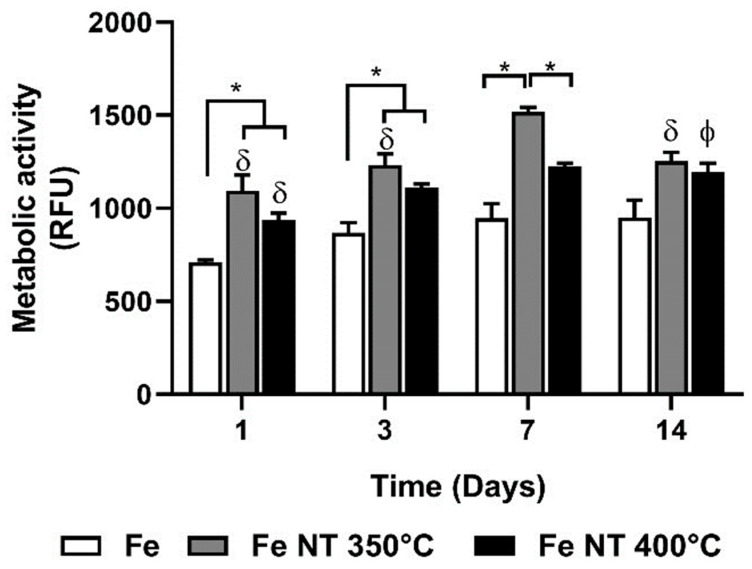
Metabolic activity of SaOs-2 cells cultured on the Fe, Fe NT 350 °C, and Fe NT 400 °C discs until 14 days of culturing. Data are displayed as mean ± stdev (*p* < 0.05, δ represents statistically different from day 7; ϕ represents statistically different from day 1; * represents statistically different from an indicated group).

The metabolic activity of SaOs-2 cells cultured onto the surface of the developed samples was investigated in vitro (Figure 9). The results showed that the cells were metabolically active during the 14 days of culture for all tested conditions, demonstrating that the discs did not have a deleterious effect on the SaOs-2 cells. This result is in agreement with those from previously reported work on in vitro cell testing that the viability and morphology of MG-63 cells were not adversely affected by extracts of nanostructured Fe oxide matrices [36]. Interestingly, cells cultured in Fe NT 350 °C and Fe NT 400 °C presented a higher metabolic activity as compared to that observed for cells grown in Fe discs. Also, while the metabolic activity of cell culture on top of Fe NT 350 °C picked up on day 7, the metabolic activity of cell culture on top of Fe NT 400 °C continuously grew over the 14 days of culture. The metabolic activity of cells cultured on top of Fe NT 350 °C decreased from day 7 to day 14, thus indicating that cells reached confluence, and their proliferation diminished [83]. On the other hand, for FE NT 400 °C, there was a linear increase as seen in the SEM and fluorescence images.

## 4. Conclusions

In the present study, pure iron samples were subjected to anodic oxidation varying the annealing temperature, which led to the following conclusions:

- It is possible to develop an ordered and uniform tubular nanostructure layer on iron oxide on the surface of pure iron. Under anodized and annealed conditions, the mean pore diameters and wall thicknesses were estimated to be 70.02 nm and 6.25 µm for Fe NT 350 °C and 60.46 nm and 5.92 µm for Fe NT 400 °C. The surface morphology of the Fe substrate before and after heat treatment revealed significant changes in surface roughness and height deviations.
- Structural characterization confirmed the predominant presence of hematite and magnetite phases in the anodized and annealed samples. The increased wettability, as evidenced by contact angle measurements, demonstrated the effect of surface modification.
- Surface modifications promoted substantial changes in cell adhesion and proliferation; therefore, when we compared the surfaces containing NTs, it was observed that on day 7, the number of cells present on the surface of Fe NT 400 °C was already greater than on the surface of Fe NT 350 °C, showing that this was the best condition, since, for the Fe NT 350 °C sample, this similarity in cell quantity was only achieved on day 14. On the other hand, the surfaces of Fe NT 350 °C showed biomineralization on Day 1, an important factor to be considered. Therefore, in vivo studies seeking to evaluate the contribution of these surfaces to cell degradation and proliferation will be decisive for choosing the calcination temperature.

## Figures and Tables

**Table 1:** XPS wide survey results for untreated Fe surfaces and Fe nanotubes annealed at 350 °C and 400 °C.

	Fe (%)	Fe NT 350 °C (%)	Fe NT 400 °C (%)
F 1s	-	2.88 ± 0.16	0.85 ± 0.12
Cu 2p	-	1.78 ± 0.50	1.74 ± 0.50
O 1s	19.64 ± 0.40	49.53 ± 0.68	50.51± 0.66
Fe 2p	1.67 ± 0.17	18.37 ± 0.52	16.55 ± 0.56
C 1s	78.69 ± 0.44	27.45 ± 0.75	30.36 ± 0.69

**Table 2:** Detected binding energy and correspondent atomic concentration of different elements on the surface of untreated and anodized samples.

Binding Energy (eV) and Atomic Concentration (%)					
	Fe 2p	O 1s	C 1s	F 1s	Cu 2p
	711.2 (0.09)				
	718.6 (0.78)	529.9 (6.97)	284.8 (64.38)		
Fe	718.8 (0.20)	531.2 (10.41)	286.0 (9.74)	-	-
	719.9 (0.09)	532.2 (2.24)	288.5 (4.64)		
	724.6 (0.47)				
	711.2 (2.75)				935.3 (0.18)
	718.6 (7.25)		283.7 (0.86)		936.9 (0.30)
	718.8 (1.94)	529.9 (33.21)	285.1 (15.07)	683.5 (0.23)	941.4 (0.24)
Fe NT 350 °C	719.5 (1.15)	531.2 (15.13)	286.6 (8.87)	683.7 (1.78)	945.6 (0.28)
	719.9 (1.30)	532.2 (1.27)	288.5 (2.71)	685.3 (0.88)	950.0 (0.22)
	724.6 (3.91)				951. 0 (0.20)
					952.7 (0.16)
	711.2 (2.42)				933.5 (0.23)
	718.6 (6.31)	529.9 (35.86)	284.8 (20.06)	683.5 (0.08)	935.2 (0.26)
Fe NT 400 °C	718.8 (1.71)	531.2 (8.11)	286.0 (8.25)	683.7 (0.42)	940.4 (0.29)
	719.5 (1.05)	532.2 (6.73)	288.5 (2.16)	685.3 (0.36)	946.0 (0.22)
	719.9 (0.95)				952.1 (0.23)
	724.6 (4.12)				952.9 (0.17)

## Data Availability

All necessary data supporting the reported results have been meticulously included in the manuscript. However, for specific requests or inquiries regarding the data, interested parties are welcome to contact the corresponding author via email. We are committed to transparency and open communication, and we will promptly address any data-related queries.
